# A 3D Fibrous Scaffold Inducing Tumoroids: A Platform for Anticancer Drug Development

**DOI:** 10.1371/journal.pone.0075345

**Published:** 2013-10-16

**Authors:** Yvonne K. Girard, Chunyan Wang, Sowndharya Ravi, Mark C. Howell, Jaya Mallela, Mahmoud Alibrahim, Ryan Green, Gary Hellermann, Shyam S. Mohapatra, Subhra Mohapatra

**Affiliations:** 1 Department of Molecular Medicine, University of South Florida, Tampa, Florida, United States of America; 2 USF Nanomedicine Research Center, Morsani College of Medicine, University of South Florida, Tampa, Florida, United States of America; 3 Chemical and Biomedical Engineering Department, University of South Florida, Tampa, Florida, United States of America; University of Texas MD Anderson Cancer Center, United States of America

## Abstract

The development of a suitable three dimensional (3D) culture system for anticancer drug development remains an unmet need. Despite progress, a simple, rapid, scalable and inexpensive 3D-tumor model that recapitulates *in vivo* tumorigenesis is lacking. Herein, we report on the development and characterization of a 3D nanofibrous scaffold produced by electrospinning a mixture of poly(lactic-co-glycolic acid) (PLGA) and a block copolymer of polylactic acid (PLA) and mono-methoxypolyethylene glycol (mPEG) designated as 3P. Cancer cells cultured on the 3P scaffold formed tight irregular aggregates similar to *in vivo* tumors, referred to as tumoroids that depended on the topography and net charge of the scaffold. 3P scaffolds induced tumor cells to undergo the epithelial-to-mesenchymal transition (EMT) as demonstrated by up-regulation of vimentin and loss of E-cadherin expression. 3P tumoroids showed higher resistance to anticancer drugs than the same tumor cells grown as monolayers. Inhibition of ERK and PI3K signal pathways prevented EMT and reduced tumoroid formation, diameter and number. Fine needle aspirates, collected from tumor cells implanted in mice when cultured on 3P scaffolds formed tumoroids, but showed decreased sensitivity to anticancer drugs, compared to tumoroids formed by direct seeding. These results show that 3P scaffolds provide an excellent platform for producing tumoroids from tumor cell lines and from biopsies and that the platform can be used to culture patient biopsies, test for anticancer compounds and tailor a personalized cancer treatment.

## Introduction

Currently, potential anticancer drugs entering clinical development have the highest level of attrition (95%)[1] of any drugs in spite of the huge amounts (∼1 billion dollars per drug) spent on their development and testing. Such a high failure rate has been partly attributed to the conventional two-dimensional (2D) monolayer cell-culture assays used for studying cancer cell biology, and screening and testing of potential anticancer drugs. In conventional 2D cell-cultures, cells grow rapidly, exhibit unnatural morphology, have lower viability, poor differentiation capability and do not show the same responses as *in vivo*.

To overcome the limitations of 2D culture, three dimensional (3D) *in vitro* tumor models are being developed [2]–[6] using a variety of biological, natural and synthetic polymers have been used to form hydrogels, films and micromolded structures in microfluidic devices and microchips [7]–[16]. 3D culture of cancer cells form multicellular aggregates termed spheroids that support anchorage-independent growth with functional and mass transport properties similar to those observed in micrometastases or poorly vascularized regions in solid tumors [17]–[22]. Despite much progress in utilizing spheroids as a screening tool for anticancer compounds, there are still several problems with the current methods of spheroid generation that limit their use as a high-throughput, robust platform. Cell manipulations on some 3D polystyrene or polycaprolactone supports require trypsinization that causes cell stress for [23]. Other platforms create artificial cell-cell or cell-matrix interactions [24], [25], have only limited mechanistic similarity to the native ECM [26], or show unstable spheroid growth [27] with central necrosis and loss of cell viability [28]. Thus, developing a 3D tumor model that more closely mimics the *in vivo* tumor microenvironment (TME) remains a formidable challenge and unmet need.

The goal of our study was to create a 3D matrix that is simple but robust and could be generated rapidly and inexpensively and would support spheroid formation of tumor cells by recapitulating *in vivo* conditions. We chose a nanoscale fiber scaffold as the platform because of the ease and reproducibility of production. Electrospinning is an excellent method for generating fibrous scaffolds of specific composition, fiber diameter and porosity from synthetic polymers such as poly(lactic-co-glycolic acid) (PLGA) and poly(e-caprolactone). The scaffold mimics the ECM in providing support and physical attachments for the spheroids. The nano- and micro-topographic and mechanotransductive cues of electrospun polymeric scaffolds [29]–[35] have been reported to stimulate migration, differentiation and gene expression of cancer cells [36]–[38]. The electrospinning conditions can be adjusted to produce fibrous scaffolds tailored for specific cell culture needs [35], [39]. Cancer cells have also been induced to form spheroids on electrospun galactosylated fibers [40] and 3D scaffolds [15]. However, 3D fibrous scaffold (3DFS)-induced tumor spheroids have been poorly characterized and it is not known whether these spheroids resemble in vivo tumors. The potential of the 3DFS as a platform for cancer drug screening has not been explored.

We have been investigating cellular differentiation on electrospun polymeric nanofiber scaffolds, and serendipitously found that cancer cells when cultured on certain polymeric 3DFS rapidly developed irregular tumor-like structures, which we are designating as ‘tumoroids’. The 3DFS, composed predominantly of PLGA random copolymer and a poly(lactide)–polyethylene glycol (PLA–PEG) block copolymer (referred to as 3P) induces self-assembly of tumor cells into tumoroids, forming a tumor/scaffold composite. We found that 3P-tumoroids mimic in vivo tumorigenesis by inducing the epithelial mesenchymal transition (EMT) that occurs during cancer metastasis. We then examined whether 3P-tumoroids could be used to test the efficacy of candidate drugs by using known anticancer drugs that are inhibitors of EMT signaling pathways. Finally, we evaluated the possibility of generating 3P-tumoroids from tumor biopsy specimens of mice and testing them for anticancer drug sensitivity. Our results show that 3P scaffolds can be used both to study the mechanism of tumorigenesis and to evaluate anticancer drugs. Our results demonstrate that the 3P scaffold may be used to generate tumoroids from patient biopsies toward developing a personalized cancer therapy.

## Results

### Preparation and characterization of the 3P scaffold

**Figure 1:**
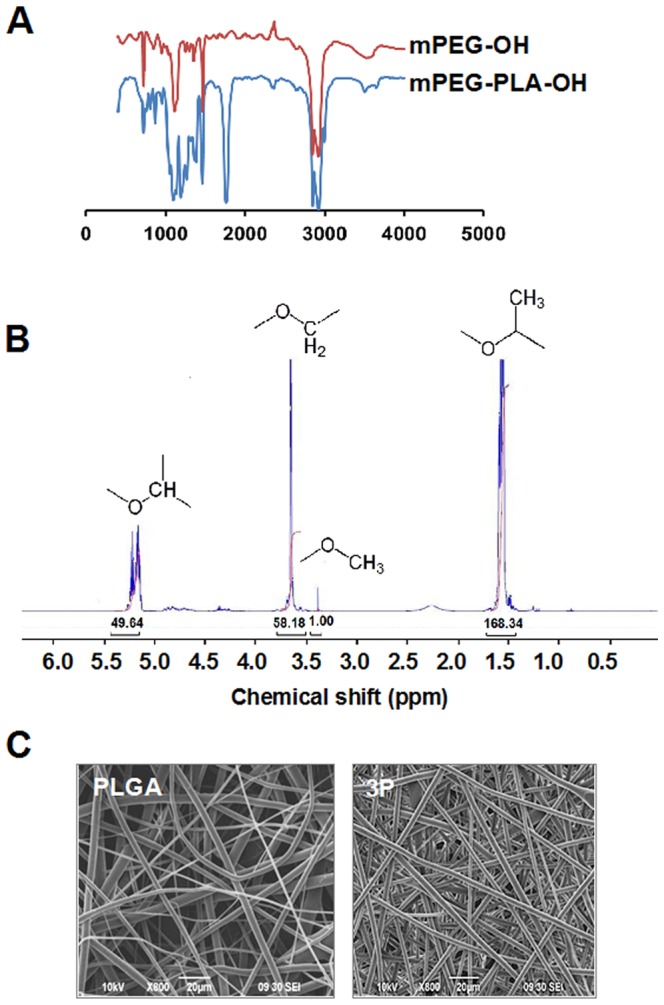
Characterization of scaffolds. (A) FTIR of mPEG and mPEG-PLA. (B) ^1^HNMR of mPEG-PLA. (C) SEM of PLGA and 3P scaffolds.

The 3P scaffolds were constructed by electrospinning a solution of the block co-polymer mPEG/LA and PLGA dissolved in appropriate organic solvents. The synthesis of mPEG/PLA was confirmed by FTIR and ^1^HNMR. FTIR shows strong absorption at 1760 cm^−1^ assigned to the –C = O stretch of PLA. The stretch of the C–O–C band of the mPEG and PLA is shown at 1087 and 1184 cm^−1^, respectively. The peaks at 2850 and 2950 represent –CH_2_ stretching of the mPEG. (Fig.1A). The molecular structure of the mPEG-PLA copolymer was characterized by ^1^H NMR (Fig.1B). The molecular weight of the PLA block of the mPEG –PLA copolymer was determined to be 23,100Da using the intensity of the terminal methoxy proton signal at 3.39 ppm as the internal standard. The scaffolds provide good spatial interconnectivity between cells, a high surface-to-volume ratio and good porosity for fluid transport. The parameters that affect the pore size, diameter and thickness of the scaffold include voltage, distance from needle tip to surface of the collecting sheet and concentration of the polymer in the solvent. Scanning electron microscopy (SEM) of the scaffold shows randomly aligned fibers that combine to form a highly porous mesh (Fig. 1C). The diameter of the fibers of the 3P scaffold ranged from 0.69 to 4.18 µm and of PLGA scaffold ranged from 0.61 to 4.95 µm with pores of mainly subcellular sizes (<10 µm).

### Formation and growth of tumoroids on 3P scaffolds

**Figure 2:**
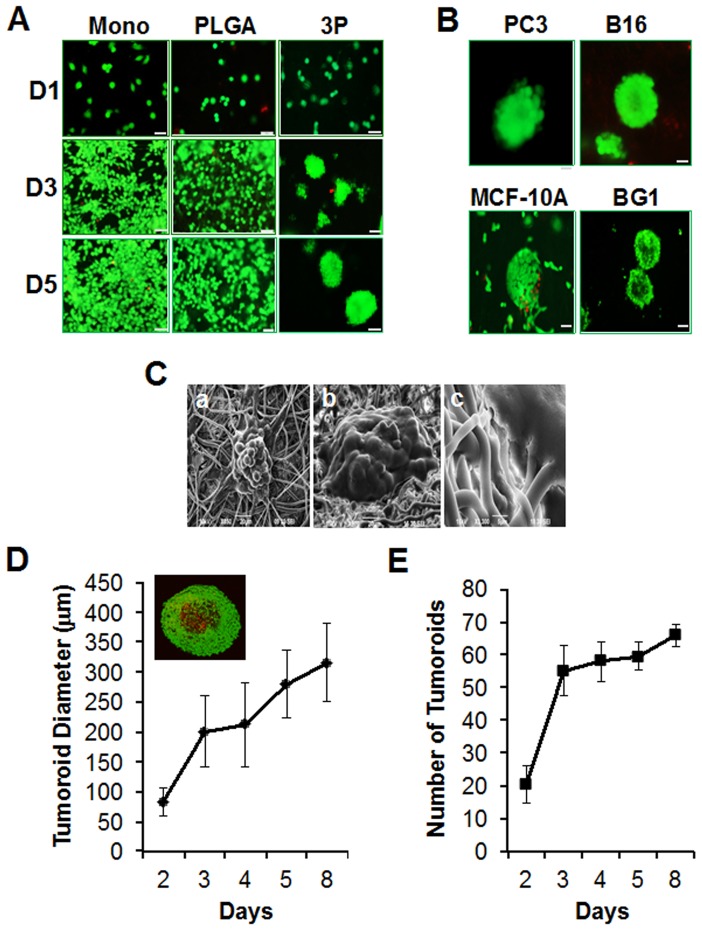
Growth of tumoroids on scaffolds. (A) LLC1 cells (5×10^3^) were cultured on monolayer, PLGA and 3P scaffolds from day 1 to day 5. Scale bar = 50 µm. (B) Growth of PC3, B16, MCF-10A and BG1 cancer cells as tumoroids on 3P scaffold. Scale bar = 20 µm. (C) SEM of LLC1 tumoroid (a) at day 3 and MCF7 tumoroid (b–c) at day7. (D) The average size of LLC1 tumoroids on 3P scaffold from day 2 to day 8. Four scaffolds/time point and 5 tumoroids/ scaffold examined. The inset shows a confocal image of a representative day 20 tumoroid. (E) Average number of tumoroids per scaffold (n = 32). Cells were stained with calcein AM/EthD-1 to detect live (green) and dead (red) cells.

LLC1 lung cancer cells were seeded on sterile scaffolds and monitored for morphology and cell viability. A comparison of cells grown on scaffolds vs. monolayer culture is shown in Fig. 2A. LLC1 cells when cultured on a 3P scaffold formed tumoroids at day three. Cells proliferated on monolayer, PLGA scaffolds or PLGA/PEG scaffolds (Fig. S1) but did not form tumoroids. Similar to LLC1 cells, PC3 prostate cancer, B16 melanoma, MCF-10A breast cancer and BG1 ovarian cancer cells also grew tumoroids on the 3P scaffolds (Fig. 2B). The SEM image shows typical tumoroid morphology with a smooth surface, tight cell junctions and indistinguishable cellular boundaries with intertwining of the fibers into and around the tumoroids that allows for anchoring and stabilization (Fig. 2C). The main parameters essential for tumoroid formation were concentration of cells and time from initial seeding of the cells. It was observed that the higher the concentrations of cells, the faster the tumoroids were formed with subsequent increase in tumoroid diameter and numbers over time (Fig. S2A). The average diameter of LLC1 tumoroids grown on a 3P scaffold at initial density of 5×10^3^ per ml for day 2 to day 8 ranged from 93 to 300 µm, respectively (Fig. 2D). The tumoroid size of 50 µm was used as the cut-off, because at this size they tend to develop due to cell-cell interactions leading to formation of tight aggregates. The average tumoroid number/ scaffold from day 2 to day 8 went from 20 to 66 (Fig. 2E). A representative composite slide showing number of tumoroids per scaffold is presented in Fig. S3. Similar growth kinetics was obtained in MCF7 cells (Fig. S2B). Typically, tumoroids exhibit a spherical proliferative geometry and beyond the diffusion capacity of oxygen and fresh growth medium the innermost cells become quiescent and die. This was observed in day 20 tumoroids that had attained diameters >500 µm (inset of Fig. 2D).

### A unique combination of topography and chemistry allows tumoroid formation on 3P scaffolds

**Figure 3:**
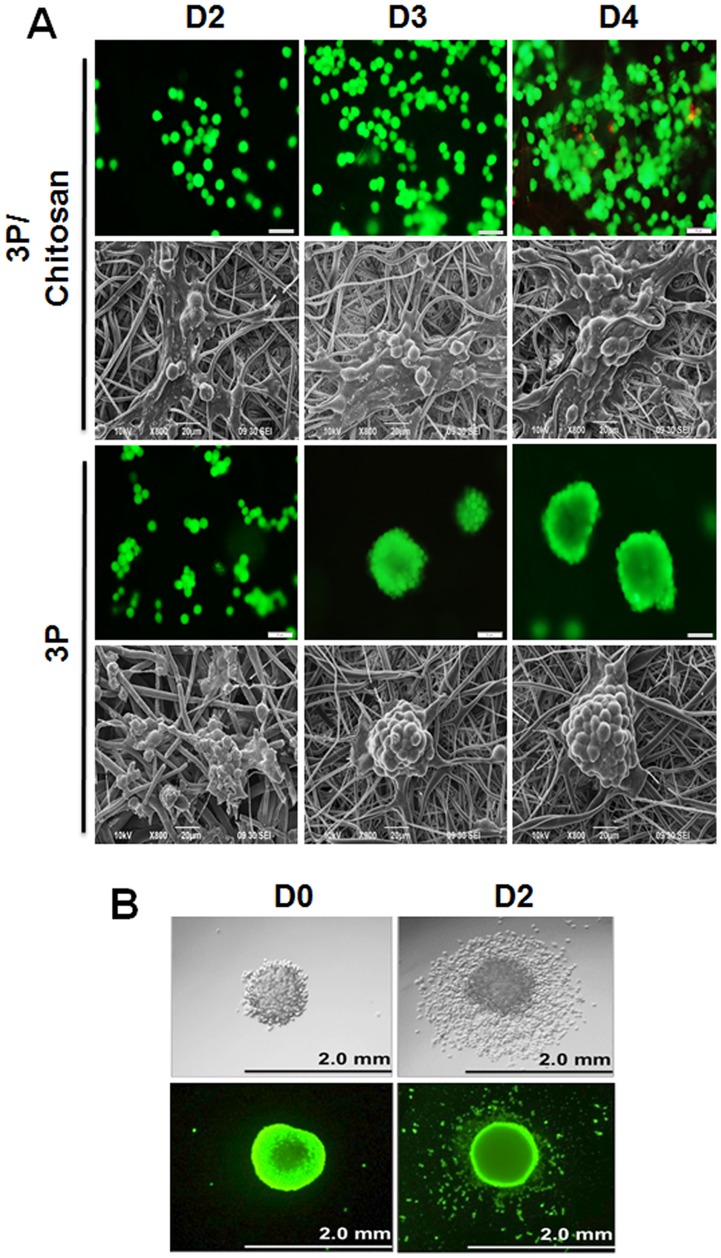
Effects of topography and surface chemistry on tumoroid formation. (A) LLC1 cells cultured on 3P scaffold and 3P/chitosan composite scaffolds for 4 days. Top panel: cells stained with calcein AM/EthD-1 to detect live (green) and dead (red) cells. Scale bar = 50 µm. Bottom panel: SEM of LLC1 cells grown on scaffold. (B) Tumoroids cultured on regular tissue culture plate. Tumoroids (day10) were transferred from the 3P scaffold onto tissue culture plate and cultured for an additional two days. Top panel: phase contrast; bottom panel: cells stained with calcein AM/EthD-1 to detect live (green) and dead (red) cells.

To explore the effects of topography on tumoroid formation, we cultured LLC1 cells on films made from polymers used to construct the 3P scaffold (Fig. S4). Cells on the 3P film formed tumoroids that easily dissociated when the substrate separated from the slide on day 5. Cells on the mPEG-PLA film grew into large disorganized aggregates that lacked the defined shape and structure of a tumoroid and also dissociated by day 5. To elucidate the effects of topography and charge on tumoroid formation we constructed a composite 3P/Chitosan scaffold. We used chitosan because it is a naturally occurring polysaccharide with a net positive charge at physiological pH thereby increasing the hydrophilic properties of the scaffold. A comparison of cells grown on 3P vs. 3P/chitosan composite scaffolds showed that LLC1 cells proliferated but did not form tumoroids on 3P (Fig. 3A). The topography of the 3P/Chitosan composite however was similar to that of 3P scaffold, as observed by SEM (Fig. 3A). Further, when LLC1 tumoroids were transferred to a regular tissue culture plate coated for monolayer culture, they adhered to the plate and migrated out from the tumoroid underscoring the importance of 3P topography in tumoroid formation (Fig. 3B). LLC-1 cells gradually migrated away from the tumoroid and ultimately formed a confluent monolayer. Tumoroids transferred to new scaffolds however maintained their morphology and shape over the same time period.

### Tumoroids grown on 3P scaffolds mimic *in vivo* tumorigenesis

**Figure 4:**
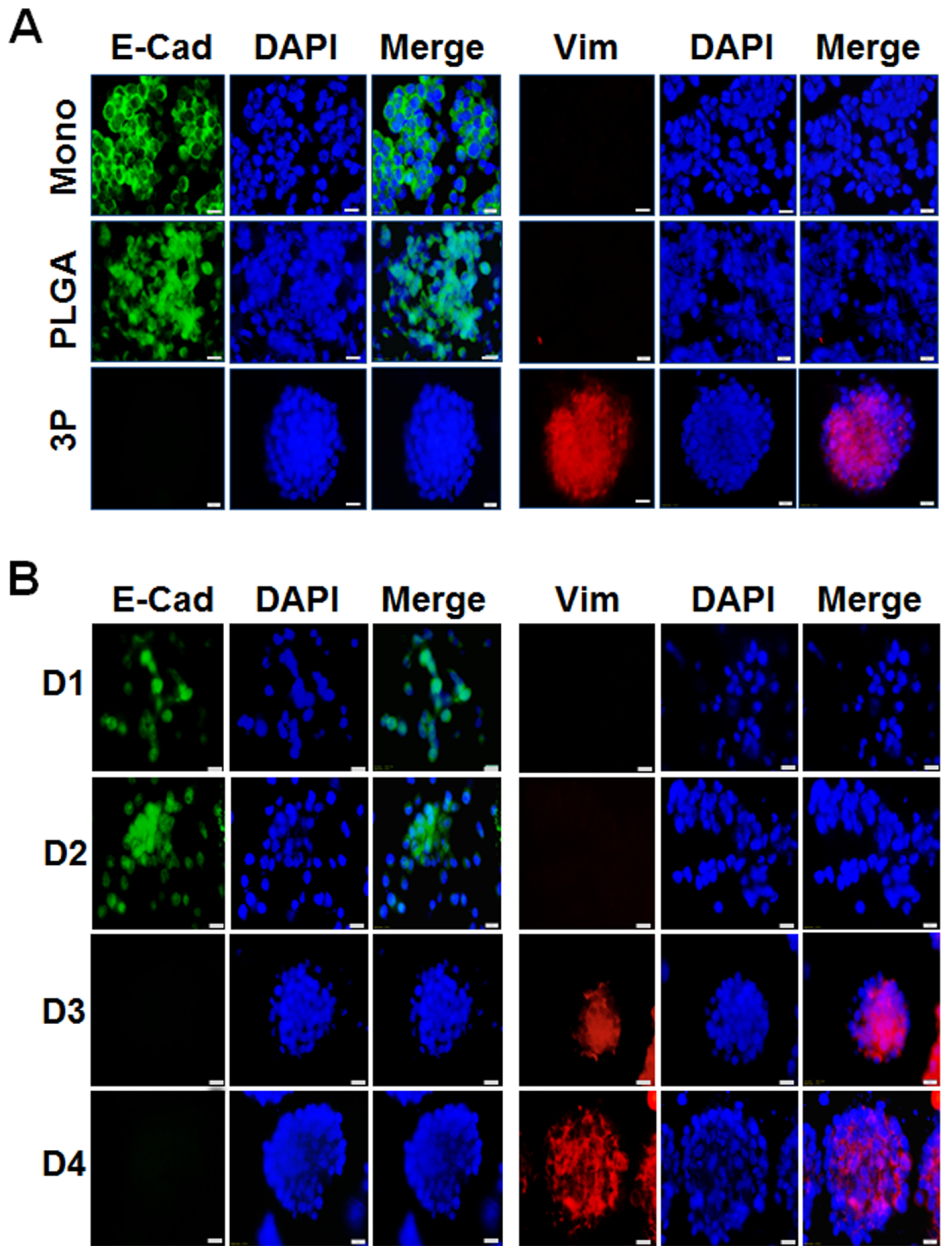
3P scaffold tumoroids showed EMT. LLC1 cells cultured on monolayer, PLGA or 3P scaffolds, fixed and immunostained with anti-E-cadherin (green), anti-vimentin (red) and DAPI (blue) for staining nuclei. (A) Tumoroids fixed at day 3; (B) Timeline of EMT induction. Scale bar = 20 µm.

Considering that 3P scaffolds induced tumor cells to form tumoroids that resemble micrometastatic tumors, we determined whether LLC-1 tumoroids formed on 3P scaffolds could undergo the EMT and compared these results with cells grown on PLGA scaffolds and in monolayer culture. At the molecular level, EMT is defined by the loss of the cell adhesion molecule E-cadherin and the transcriptional induction of the mesenchymal marker vimentin [41], [42],[43], [44]. Upon culturing on 3P scaffolds, LLC1 cells formed tumoroids at day 3 that expressed vimentin while none was observed in the monolayer culture or in cells cultured on a PLGA scaffold (Fig. 4A). Additionally tumoroids exhibited a loss of E-cadherin expression on 3P, while cells cultured as a monolayer or on a PLGA scaffold retained this expression. Similar to LLC1 cells, B16, BG1, MCF7, MDA-MB231 and PC3 cells gained mesenchymal phenotype when cultured on the 3P scaffold (Fig. S5). This suggests that tumoroid formation may correlate with enhanced invasive potential and tumorigenicity, characteristics that define the EMT and that appear to be manifested within the context of the 3P environment. To determine the timeline of EMT induction, LLC1 cells were cultured on a 3P scaffold and stained for vimentin and E-cadherin from day 1 to day 4. It was observed that the onset of EMT occurred at day 3 and continued into day 4 correlating with the self-assembly of the cells into tumoroids (Fig. 4B).

### Altered sensitivity of tumoroids to EMT-specific inhibitors

**Figure 5:**
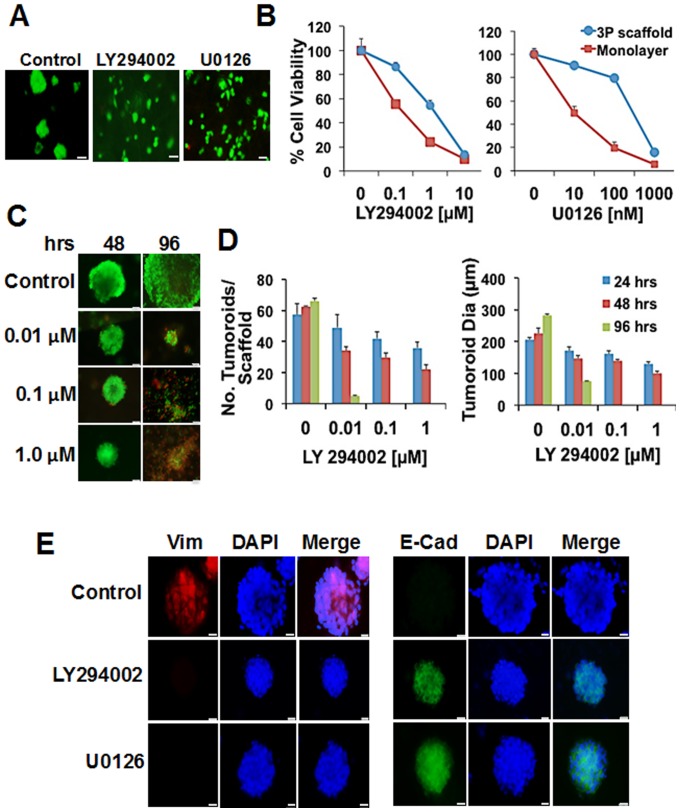
Altered sensitivity of tumoroids to EMT specific inhibitors. (A) The PI-3K inhibitor (LY294002) and MAPK inhibitor (U0126) prevented MCF7 tumoroid formation on 3P scaffold. Scale bar = 50 µm. (B) Dose-dependent cytotoxic response of MCF7 cells cultured on the 3P scaffold vs. monolayer. (C–D) Inhibitors induced cell death in LLC1 tumoroids. LLC1 tumoroids (day 4) were exposed to various concentrations of LY29400 for the indicated days and then stained with calcein AM/EthD-1 and observed for changes in tumoroid size and numbers. Four scaffolds/time point and 5 tumoroids/scaffold examined. The experiments were repeated in triplicates and data presented as mean ± SD. * p<0.05. Scale bar = 50 µm. (E) Abrogation of EMT in LLC1 tumoroids treated with inhibitors. Cells were fixed and immunostained using anti-E- cadherin (green), anti-vimentin (red) and DAPI (blue). Scale bar = 20 µm.

To understand whether EMT plays a role in tumoroid formation, we examined the effects of known antitumor agents as control modulators of EMT [45] [46], [47]. Both mitogen activated protein kinase (MAPK) and phosphatidylinositol-3 kinase (PI3K) signaling pathways are known regulators of EMT. Hence, we used U0126 that selectively inhibits MEK, a dual specificity kinase in the MAPK cascade from phosphorylating ERK1/2, and LY294002 that inhibits the PI3K pathway by dephosphorylation of AKT, as specific EMT inhibitors. First, to evaluate if treatment can prevent tumoroid formation, MCF7 cells were cultured on 3P scaffolds in the presence or absence of LY294002 or U0126. Both inhibitors prevented tumoroid formation (Fig. 5 A) and reduced cell viability with increasing dose of inhibitors (Fig. 5B). Scaffold-grown MCF7 cells showed reduced sensitivity to inhibitors compared to cells on monolayer, as determined by the IC-50 (Fig. 5B). The IC-50 of LY294002 for the monolayer was 0.1 µM and for the 3P scaffold was 1.092 µM. Similarly, the IC50 of U0126 on the monolayer and 3P scaffold were 6.72 nM and 652 nM, respectively.

Our second approach was to determine if these inhibitors were effective in modulating tumoroid growth. We treated LLC1 cells after 48 hours of culture on the 3P scaffold with LY294002 or U0126 and monitored tumoroid formation (Fig. 5 C–D). Measurements of tumoroid size and numbers revealed a dose-dependent cytotoxicity in treated tumoroids compared to untreated tumoroids. With increasing concentration of drugs, we observed more cell death accompanied with the dissolution of the tumoroids. At 24 hours after treatment, tumoroids treated with LY294002 at concentration of 0.01–1 µM demonstrated an average decrease of 17 to 37% in size respectively compared to untreated tumoroids. At 48 hours after treatment tumoroid size decreased on average 29 to 51%, respectively and at 96 hours after treatment, cells treated with 0.01 µM decreased 63% while those treated with 0.1 µM and 1.0 µM of inhibitor were found dead. Tumoroid numbers also decreased substantially by 14% at 24 hr and 93% at 96 hrs post-treatment with tumoroids treated with 0.1 µM and 1.0 µM of inhibitor dissipated at 96 hours post treatment.

To determine if treatment with these inhibitors inhibited EMT in fully formed tumoroids, we administered the same concentration of drugs to tumoroids cultured at day 4 on the 3P scaffold. Results showed that both LY294002 and U0126 inhibited vimentin expression while inducing expression of E-cadherin similar to that observed with cells cultured on PLGA scaffolds alone and on monolayer (Fig.5E). These findings implicate both PI3K and the MAPK signaling as significant pathways in tumoroid formation and EMT expression on 3P scaffolds.

### Chemosensitivity of tumor biopsies grown on 3P scaffolds

**Figure 6:**
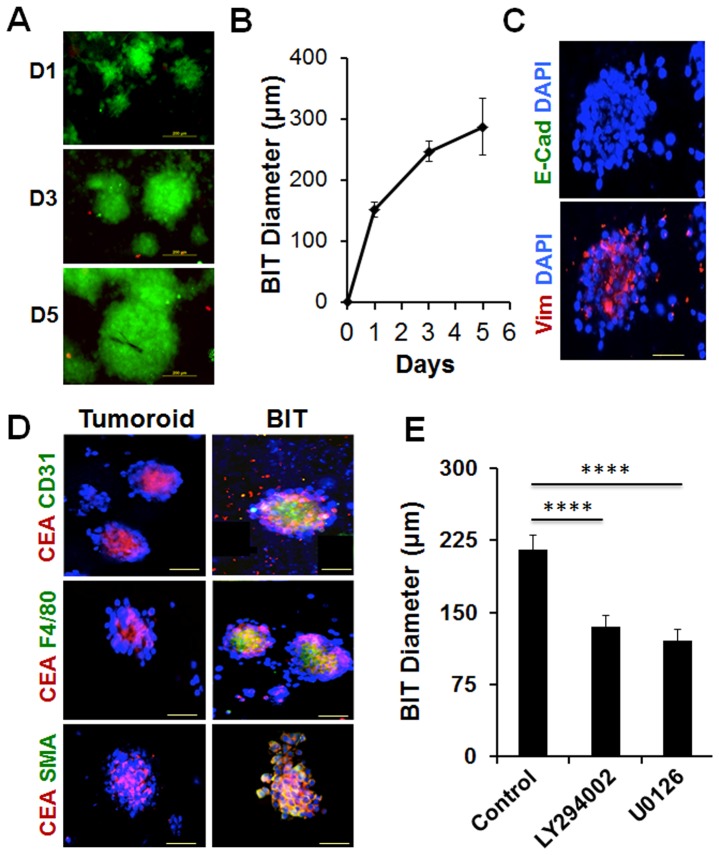
Culturing tumor biopsies on 3P scaffold. (A–B) FNA of implanted LLC1 mouse tumors were cultured on 3P scaffolds for the indicated time. Cells were stained with calcein AM/EthD-1 (A). The average size of BITs on 3P scaffolds from day 1 to day 5 is shown (B). (C) BITs were fixed and immunostained for E-cadherin (green), vimentin (red) and DAPI (blue). (D) LLC1 tumoroids and BITs were dual-immunostained for CEA (red) and either CD31, F4/80 or SMA (green) and DAPI (blue). Scale bar = 100 µm. (E) BITs (day 2) were treated with 1 µM each of LY294002 or U0126 for 3 days. The average size of BITs on 3P scaffolds (n = 10 controls, 55 LY294002 and 50 U0126) shown. Scale bar = 200 µm.

To determine if the 3P scaffold can be utilized to culture tumor biopsies, single-cell suspensions of FNAs of implanted mouse tumors were cultured on 3P scaffolds. As shown in Fig. 6 A–B, the average diameter of biopsy induced tumoroids (BITs) increased from day 1 to day 5 at approximately the same rate as observed in the experiments with tumor cell lines. Immunostaining of BITs showed loss of expression of E-cadherin and gain of expression of vimentin suggesting EMT induction (Fig. 6C). In addition to tumor cells, BITs were examined for the presence of stromal cells that are typical of in vivo tumor microenvironment (TME) [48] by immunostaining with antibodies to respective cell surface markers. BITs were found positive for tumor cell marker, carcinoma embryonic antigen (CEA), macrophage marker F4/80, endothelial progenitor cell marker CD31 and cancer associated fibroblast marker smooth muscle actin (SMA), indicating the presence of stromal components of the TME (Fig. 6D). LLC1 tumoroids, used as control expressed only CEA.

To determine if the biopsy tumoroids cultured on 3P scaffolds can be utilized to assess drug sensitivity, FNA tumoroids were cultured in the presence or absence of the inhibitors, LY294002 and U0126. Results showed that inhibitors were effective in modulating growth of BITs as revealed by decrease in tumoroid size and a dose-dependent cytotoxicity. However, the IC-50s of LY294002 and U0126 were >1 µM (Fig. 6E) indicating a greater resistance of BITs to the drugs than LLC1 tumoroids. Increased drug resistance of biopsy tumoroids in the 3P system was not due to a lack of drug uptake.

## Discussion

In this study, we report on a novel 3P fibrous scaffold that induced the formation of a micrometastatic compact aggregate of tumor cells that we term a ‘tumoroid’. Our platform has several advantages over existing 3D technologies. (1) The 3P fibrous scaffold is produced from FDA-approved synthetic polymers. (2) It is conveniently and cheaply manufactured by electrospinning, which creates a mat of randomly distributed nano- to micro-scale fibers and can permit scale up production. (3) The scaffold mats can be cut into smaller pieces for placement in the wells of standard plastic cell-culture dishes. (4) Cancer cells seeded onto these scaffolds with growth medium containing the appropriate factors grow as tumoroids that show the EMT characteristic of *in vivo* tumorigenesis. (5) This platform allows the coculture of tumor cells and stromal cells to identify changes in specific factors, gene expression and invasion potential. Tumoroids are able to respond to the same biochemical, nanotopographical and mechanical cues that drive tumor progression in the native ECM. (6) The 3P platform can be used to evaluate therapeutic strategies for simultaneously targeting tumor cells and the stromal cells that are components of the stem cell niche. (7) The 3P platform can also be adapted for use in a point-of-care device to culture a patient's own cells from a biopsy, test them *ex vivo* with different anticancer compounds and tailor a personalized treatment.

To create a 3D surface that mimics the *in vivo* tumor microenvironment we utilized electrospinning to produce a 3P scaffold that induces cancer cells to develop into tumoroids by mechanisms that are presently unknown. There are two possibilities: (i) cell-cell interaction leading to tight aggregates that form the tumoroids, and (ii) outward proliferation of a fiber-attached cell to form a tumoroid. These possibilities are not mutually exclusive. Electrospun fibrous mats mimic ECM structures and the pore size, diameter and thickness of the fibers may regulate cell behavior [49]. Further, scaffold topography and architecture have been shown in previous studies to influence tumor cell behavior [37], [38], [50]–[53]. Our 3P scaffold is composed of random fibers of a mixture of PLGA and PLA-PEG block-copolymer. While PLGA and PLA possess good mechanical properties, controlled degradability, and excellent biocompatibility [54]–[56], PEG alters the electrostatic binding properties of cells and promotes cell-cell interactions leading to assembly of tumoroids [57]–[60]. Although not fully elucidated, these characteristics may explain the spatial and temporal forces required to trigger tumoroid formation on the 3P scaffold. In addition, the scaffold provides good spatial interconnectivity between cells, a high surface-to-volume ratio and good porosity for fluid transport. It mimics the physical interaction of the tumor with the topographical features of the native ECM defined by the presence of nano-micro sized fiber and sub cellular pores.

To elucidate the effects of topography, we cultured LLC1 cells on films made from polymers used to construct the 3P scaffold. LLC1 cells cultured on the 3P scaffold formed tumoroids that persisted, while cells cultured on the same polymer deposited as a film formed tumoroids that quickly disassociated. We also found that similar-sized scaffolds of PLGA only do not form tumoroids, underscoring the contribution of PEG-PLA block copolymer in the fiber, which by itself is not amenable to electrospinning. To understand the effects of charge on tumoroid formation we changed the scaffold surface from a neutral charge to a positive charge by surface coating with cationic chitosan; this would increase the hydrophilicity and improve cellular interactions with scaffold. However LLC1 and MCF7 cells proliferated on the scaffold but did not form tumoroids or undergo EMT (Fig. S6). This suggests that charge in addition to topography plays an important role in tumoroid formation. Moreover, tumor cells migrated from 3P tumoroids that had been transferred from the scaffold to tissue culture plates. These results showed that chemical and physical cues from the 3P scaffolds contributed to tumoroid formation.

Others have shown that deprivation of glutamine [61] or culturing cells on hard [62] and soft agar [63] resulted in selection of highly adaptable metastatic cancer cells. These reports suggest that the topography of 3P scaffolds may be forcing a selection of structural adaptability, potentially promoting EMT, cancer stem cell-like features and chemotherapy resistance. Although the precise mechanism whereby the scaffold induces tumoroid formation is unknown, the results of our studies on tumoroids taken together do not support this notion because of the following. First, as described above, tumoroids arise as a result of tight aggregation of cells, not because of clonal proliferation. Second, as opposed to adaptive selection of tumor cells, which takes longer time and generates spheroids in very low frequency, the tumoroids arise quickly-within three days after plating-and at a high frequency (∼70%). Thus, while the details of the contribution of topography remain unknown, the random fiber topography appears to promote cell-cell, not cell-matrix interaction leading to formation of tight aggregates and then tumoroids exhibiting EMT.

All of the tumor cell lines examined developed tumoroids on 3P scaffolds and induced EMT with a loss of E-cadherin expression, a condition that imparts invasive and migratory capacity to cells and poor prognosis in several cancers [41], [42]. Concomitant with down-regulation of E-cadherin was the expression of vimentin, a major cytoskeletal protein ubiquitously expressed in mesenchymal cells and tumor cells undergoing metastasis [43], [44]. Tumor cells cultured as monolayers or on unmodified PLGA scaffolds maintained epithelial marker expression and did not undergo EMT. The majority of *in vitro* studies of EMT involve cultured cells that are induced either by forced expression of selected transcription factors or prolonged exposure to inducers such as growth factors and cytokines, whereas in our experiments the EMT occurred as a consequence of tumoroid formation. The EMT is considered an early event in the metastatic process and studies have suggested that dissemination may occur prior to tumor development [64], [65]. In most experimental systems, a complete change in EMT marker expression requires ten days or longer [66]. Expression of EMT markers by LLC1 tumoroids on the 3P scaffold occurred at day three of culture. The 3P scaffold may supply physicochemical cues that trigger the EMT by altering cell-cell and cell-matrix interactions, promoting architectural reorganization, or affecting the binding of proteins to cell surface receptors involved in signal transduction pathways [57]–[60]. It has been shown that pathways involving PI3K/AKT, MAPK and TGF-β communicate via growth factors and cytokines to repress E-cadherin and up-regulate mesenchymal genes during EMT [45]. TGF-β is the major inducer of EMT and acts through canonical and noncanonical pathways to affect PI3K/ AKT, MAPK signaling [46], [47]. In support of this observation, we found that treatment of cancer cells with LY294002 (PI3K inhibitor) and U0126 (MAPK inhibitor) prevented tumoroids underscoring the importance of EMT in *in vitro* tumoroid formation.

Treatment of tumoroids with LY294002 or U0126 on 3P scaffolds vs monolayer culture showed higher drug resistance of MCF7 cells on the scaffold. The IC-50 values for 3P cultures were 10 fold higher for LY294002 inhibitor and 100 fold higher for U0126 inhibitor than for monolayer cultures. These results are supported by other studies showing that tumor cells grown as 3D tumoroids develop multicellular resistance to most cytotoxic drugs compared to those grown in monolayer culture [67], [68]. For example, the *in vivo* drug-resistant variant, EMT-6, of mouse mammary tumor cells lost resistance when cultured as a monolayer but regained it when regrown *in vivo* as a solid tumor or *in vitro* as spheroids [69]. The reasons for the differences between 3D and monolayer cultures remain unknown. Pathophysiological gradients to nutrients, oxygen and drugs and the concentric arrangement of heterogeneous cell populations within the 3P tumoroids may affect RNA and protein expression. For example, it has been reported that quiescent cells near the necrotic core of a tumoroid up-regulate the cyclin-dependent kinase inhibitor p27Kip1, which arrests cells in the G0/G1 phase and induces cell cycle resistance [70]. The expression of p27Kip1 is fifteen times greater in tumoroid culture than in monolayer culture. In addition, inhibition of apoptosis via the Bcl-2 pathway [71], and the down-regulation of PMS2 and topoisomerase 11 DNA mismatch repair proteins [72], [73] have been proposed as mechanisms that desensitize tumor cells in spheroids to antitumor agents. We showed that the resistance of tumoroids on 3P scaffolds to anticancer agents was not a result of a mass transport defect. This is consistent with other drug penetration data that have shown that despite efficient drug penetration, tumoroids still exhibit higher drug resistance than monolayer cultures [74], [75]. These data demonstrate that tumoroid growth on 3P scaffolds reflects physiological growth conditions in which changes in cell phenotype and signaling add significant cues for the regulation of cell behavior and drug resistance.

Tumor-stroma interactions affect cancer growth, differentiation, metastasis and the acquisition of drug resistance. To determine if the 3P scaffold was an appropriate *in vitro* model for anticancer drug screening we cultured tumor biopsy specimens on the scaffold. 3P scaffolds supported the growth of both tumor and stromal cells from the biopsy and this *in vitro* platform therefore mimics *in vivo* tumor growth. Moreover, tumoroids derived from the biopsies (BITs) were more resistant to MEK and PI3K inhibitors than the tumoroids from cancer cell lines. Thus, while tumoroids from a cell line exhibit a distinct drug sensitivity profile, they do not accurately reflect the *in vivo* condition. Our biopsy results suggest that co-cultures of tumor cells with stromal cells will produce tumoroids that better reflect the *in vivo* context. These tumor/stroma effects on chemosensitivity underscore the importance of using a 3D platform such as the 3P scaffold for growth of actual patient tumor biopsies to test the efficacy of anticancer drugs.

Our scaffold-based platform provides an elegant approach for developing customized anticancer treatments. Tumor biopsies can be cultured on a 3P scaffold, differentiated into BITs and screened with an array of anticancer drugs to determine the most effective drug combinations for a particular cancer patient within a week. Rapid advances in genetics, genomics and related technologies are promising a new era of personalized cancer therapy based on molecular characterization of a patient's tumor and its microenvironment with the intent to improve outcomes and decrease toxicity [76]. However, these molecular predictors of tumor response are far from perfect. The heterogeneity of tumors, the lack of effective drugs against most genomic aberrations and the technical limitations of molecular tests hamper the current approaches for predicting the response to anticancer drugs. Our novel 3P scaffold may provide the solution for overcoming these hurdles to rapid drug screening and customized cancer therapy.

## Materials and Methods

### Synthesis of mPEG-PLA copolymer

Methoxy PEG-PLA block copolymer was prepared by ring-opening polymerization. Briefly, 3,6-dimethyl-1, 4-dioxane-2, 5-dione (LA)(Fisher) was dried in a vacuum oven at 40°C overnight. 1 g of mono-methoxy poly(ethylene glycol) (mPEG; Sigma-Aldrich) was flame dried in a 100 ml three-necked round-bottom flask and stirred at 80°C for 2 hours under vacuum. 4 g of dried LA polymer and 0.2 wt% stannous octoate (Sn(Oct)_2_)(Sigma-Aldrich) were added to the flask under the protection of argon gas. The mixture was dissolved in 20 ml anhydrous toluene and heated at 140°C under argon gas for 5 hours. Solid products of the diblock copolymers were obtained by adding the polymer solution to ice cold diethyl ether. The product were dissolved in dichloromethane and precipitated in cold diethyl ether twice, for purification. The final copolymer was dried in a vacuum oven at 50°C for 48 hours. The prepared polymer was characterized by FTIR using a Nexus spectrometer and ^1^HNMR using a Bruker 250 spectrometer.

### Construction of the 3P Scaffold or film

To construct the 3P scaffold, 1 g of poly(lactic co-glycolic acid) (Sigma-Aldrich) and 3 g of mPEG-PLA polymer were dissolved in a solution of dichloromethane and chloroform (80/20 v/v). For the construction of the PLGA scaffold a 3%(w/v) of PLGA in a solution of dichloromethane and chloroform (80/20 v/v) was used. The solutions were electrospun at a positive voltage of 20kVDC and a flow rate of 0.2–0.5 ml/hr using a high voltage power supply (Gamma High Voltage Research, USA) and a syringe pump (kD Scientific). The fibers were collected on an aluminum covered copper plate at a fixed distance of 20 cm. The scaffolds were cut to approximately7×7 mm^2^ and placed in 96 well plates, sterilized in isopropyl alcohol, washed three times in PBS then additionally sterilized by exposure to high intensity UV light for one hour. The diameter and pore size of the scaffolds were determined from images obtained from a scanning electron microscope (Jeol JSM 6490).

PLGA/mPEG-PLA or mPEG-PLA films were prepared by casting the same formulation of 3P scaffold solution onto glass slides and allowed to dry. The mPEG or chitosan coated PLGA scaffold were constructed by immersion of PLGA scaffold in 1% of mPEG aqueous solution or 1% chitosan (deacylation>95%) in 1% acetic acid solution for four days. For the migration assay, day 10 tumoroids were transferred from the 3P scaffold to a tissue culture plate containing complete medium and the migration was monitored.

### Tumoroid diameter and number estimation

MCF-10A and MCF7 breast cancer, PC3 prostate cancer, B16 melanoma, BG1 ovarian and LLC1 Lewis lung cancer cells. All cell lines except BG1 were purchased from ATCC. BG1 cells [77] were kindly provided by Dr. Wenlong Bai, University of South Florida. All cells were grown in Dulbecco's modified Eagle's medium (DMEM) (GIBCO) supplemented with 10% fetal bovine serum (FBS) (Atlanta Biologicals) 1% (v/v) penicillin and streptomycin (GIBCO). Cells were maintained in a 37°C humidified incubator with an atmosphere of 5% carbon dioxide/95% air. Single-cell suspensions were prepared by treatment with trypsin-EDTA (GIBCO) and resuspended in complete medium before monolayer or tumoroid culture was set up. Cells from a single-cell suspension were added to scaffolds in 96 well plates in a total volume of 100 µl. Tumoroids were allowed to form under these conditions from three to eight days. For measurement of tumoroid diameter, at least five fluorescent images were taken randomly per scaffold with at least one tumoroid captured per image. To compensate for variation in radii, two diameters representing the shortest and longest diameters were drawn through the center of the tumoroid and the final diameter was calculated as the average of both values using Image J software. The presented diameter was the average of 20 tumoroids/day. Tumoroid numbers were counted from full planar images taken systematically across each scaffold. Rounded aggregates of size ≥50 µm were counted as tumoroids. For longer term culture greater than eight days, scaffolds were transferred to larger wells to accommodate demanding nutrient requirements. Larger tumoroids >600 µm can be easily detached from the scaffold using gentle pipetting and transferred to new scaffolds. Tumoroids and monolayer cells were stained with calcein AM/Ethd-1 (Invitrogen) that stains live cells green and dead cells red. The scaffolds were placed on glass slides, covered with glass coverslips then viewed under a fluorescence microscope (Olympus BX51) or a confocal microscope (Leica TCS).

### Scanning Electron Microscopy

LLC1 cells (5×10^3^) and MCF7 cells (7×10^3^) were cultured on scaffolds and allowed to form tumoroids at day 3 or 7 days respectively. The scaffolds were fixed in a 50∶50 (v/v) solution of 2.5% glutaraldehyde in 0.2 M cacodylate buffer (pH 7.1) for 24 hrs. The scaffolds were washed in 1% osmium tetroxide in cacodylate buffer at 40°C for one hour. The scaffolds were further dehydrated in an ascending series of ethanol at concentrations 10%, 35%, 50%, 70%, 95% and 100% for ten minutes. Final dehydration was done in hexamethyldisilazane (HMDS) for 10 minutes. Samples were air dried then sputter-coated with gold at a voltage of 20MA for 30 seconds under argon gas. The tumoroids were viewed on a Jeol JSM 6490 scanning electron microscope. All reagents were purchased from Electron Microscopy Sciences.

### Immunofluorescence microscopy

Tumoroids cultured on the 3P scaffold were washed three times with PBS then fixed with 4% paraformaldehyde in PBS for 20 minutes at room temperature, followed by permeabilization in 1.0% Triton X-100 for 15 minutes. The tumoroids were incubated with 1% BSA for 30 mins at room temperature. Tumoroids were incubated with the primary antibody overnight at 4°C, washed three times with PBS then incubated with the secondary antibody for one hour at room temperature in the dark. Vimentin (Cell Signaling), and E-cadherin (Santa Cruz Biotechnology) antibodies were used at 1∶200 dilution and all antibody solutions were prepared in 1% BSA in PBS. Secondary antibodies were used at 1∶100 dilution. Dual immunostaining was performed as described by Mallela et.al. [48]. CEA antibody was used at 1∶200, CD31 at 1∶50 and F480 and SMA at 1∶100 dilutions.

### Drug sensitivity of cells on 3P scaffold

MCF7 cells were cultured in 3P scaffolds at a cell density of 7×10^3^/scaffold for two days in complete medium. The culture medium was carefully removed and replaced with 100 µl of fresh medium containing varying concentrations of PI-3K inhibitor LY294002 or MAPK inhibitor U0126 (Cell Signaling). After treatment, the cells were incubated for three days in a humidified atmosphere under 5% CO_2_ at 37°C then stained with calcein AM/ Ethd-1 and observed for tumoroid formation. The viable cell number was counted using Image J software with calcein AM staining. The cell viability was estimated by dividing the treated viable cells by untreated viable cell number. The IC-50, the concentration of drugs required to inhibit 50% cell growth was calculated from the dose response curve using GraphPad Prism Software (version 5.01).

For evaluating effects of inhibitors on established tumoroids, LLC1 cells were seeded in the 3P scaffolds at a cell density of 5×10^3^/scaffold and cultured for four days to allow for tumoroid formation. The culture medium was replaced with fresh medium containing various concentrations of LY294002 and U0126. The medium was replaced by fresh medium or medium containing drug every two days. Tumoroids were stained with calcein AM/ Ethd-1 at day 2 and day 4 post-treatment and evaluated for tumoroid size and number.

### Culturing fine needle aspirates (FNAs) of implanted mouse tumors

LLC1 cells (5×10^5^) were subcutaneously injected into the flanks of wild type C57BL/6 mice (National Cancer Institute). Tumor formation was monitored for 2 weeks after which tumor biopsies were collected by FNAs. Briefly, a 23 ga needle was inserted into the tumor using a rotating motion and the tissue samples were flushed from the needle by attaching a syringe filled with tissue culture medium and expelling the contents into a sterile tube. This process was repeated twice. The three collected tumor samples were pooled and single cell suspensions were cultured at on the scaffold.

### Ethics Statement

All animal studies were approved and conducted according to guidelines of the University of South Florida Institutional Animal Care and Use Committee.

### Statistical analysis

Quantitative data were expressed as mean +/− standard deviation. Student's t- test was used to analyse the statistical significance of the data between groups. Values with p<0.05 were considered statistically significant. All experiments were performed in triplicates.

## Supporting Information

Figure S1 **Scaffold composition for tumoroid formation.** LLC1 cells were cultured on PLGA, PLGA/PEG and 3P scaffold and stained with calcein AM/EthD-1 to detect live (green) and dead (red) cells. Scale bar = 50 µm.
(TIF)

Figure S2 **The relationship between seeding density and tumoroid formation.** (A) LLC1 cells were cultured at concentrations ranging from 3×10^3^ to 1×10^4^. (B) MCF7 cells were cultured at 7×10^3^ to 15×10^3^. Cells were stained with calcein AM/EthD-1 to detect live (green) and dead (red) cells. Scale bar = 50 µm.
(TIF)

Figure S3 **Full planar distribution of LLC1 tumoroids on 3P scaffold (day3).** A representative composite picture of all sectors of the scaffold viewed using a10× lens. Scale bar = 500 µm.
(TIF)

Figure S4 **Effects of topography on tumoroid formation.** LLC1 cells (5×10^4^) were cultured on 3P and mPEG-PLA films and on 3P scaffold (5×10^3^) from days 3–5. Cells were stained with calcein AM/EthD-1 to detect live (green) and dead (red) cells. Scale bar = 500 µm.
(TIF)

Figure S5 **Cancer cells grown on 3P scaffold showed EMT.** B16, BG1, MCF7, MDA-MB231 and PC3 cells cultured on 3P scaffolds or monolayer, fixed and immunostained with anti-E-cadherin (green), anti-vimentin (red) and DAPI (blue) for staining nuclei. Scale bar = 20 µm.
(TIF)

Figure S6 **LLC1 grown on 3P/chitosan composite scaffold failed to show EMT.** LLC1 cells cultured on 3P/chitosan composite scaffold or 3P scaffold, fixed and immunostained with anti-E-cadherin (green), anti-vimentin (red) and DAPI (blue) for staining nuclei. Scale bar = 20 µm.
(TIF)
