# Supplementary figures and images for: A 3D Fibrous Scaffold Inducing Tumoroids: A Platform for Anticancer Drug Development
Source: PLoS One. 2013 Oct 16;8(10):e75345. doi: 10.1371/journal.pone.0075345 (PMC3797770; doi:10.1371/journal.pone.0075345)

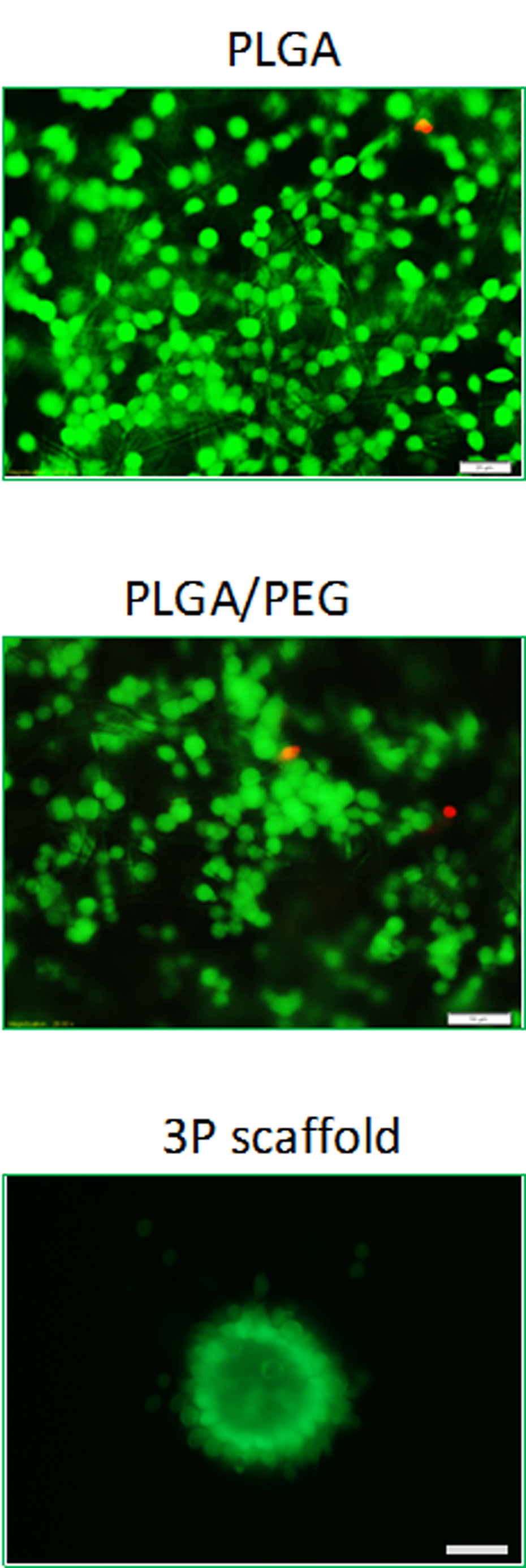

Supplement: Figure S1 — Scaffold composition for tumoroid formation. LLC1 cells were cultured on PLGA, PLGA/PEG and 3P scaffold and stained with calcein AM/EthD-1 to detect live (green) and dead (red) cells. Scale bar = 50 µm. (TIF) [file pone.0075345.s001.tif]

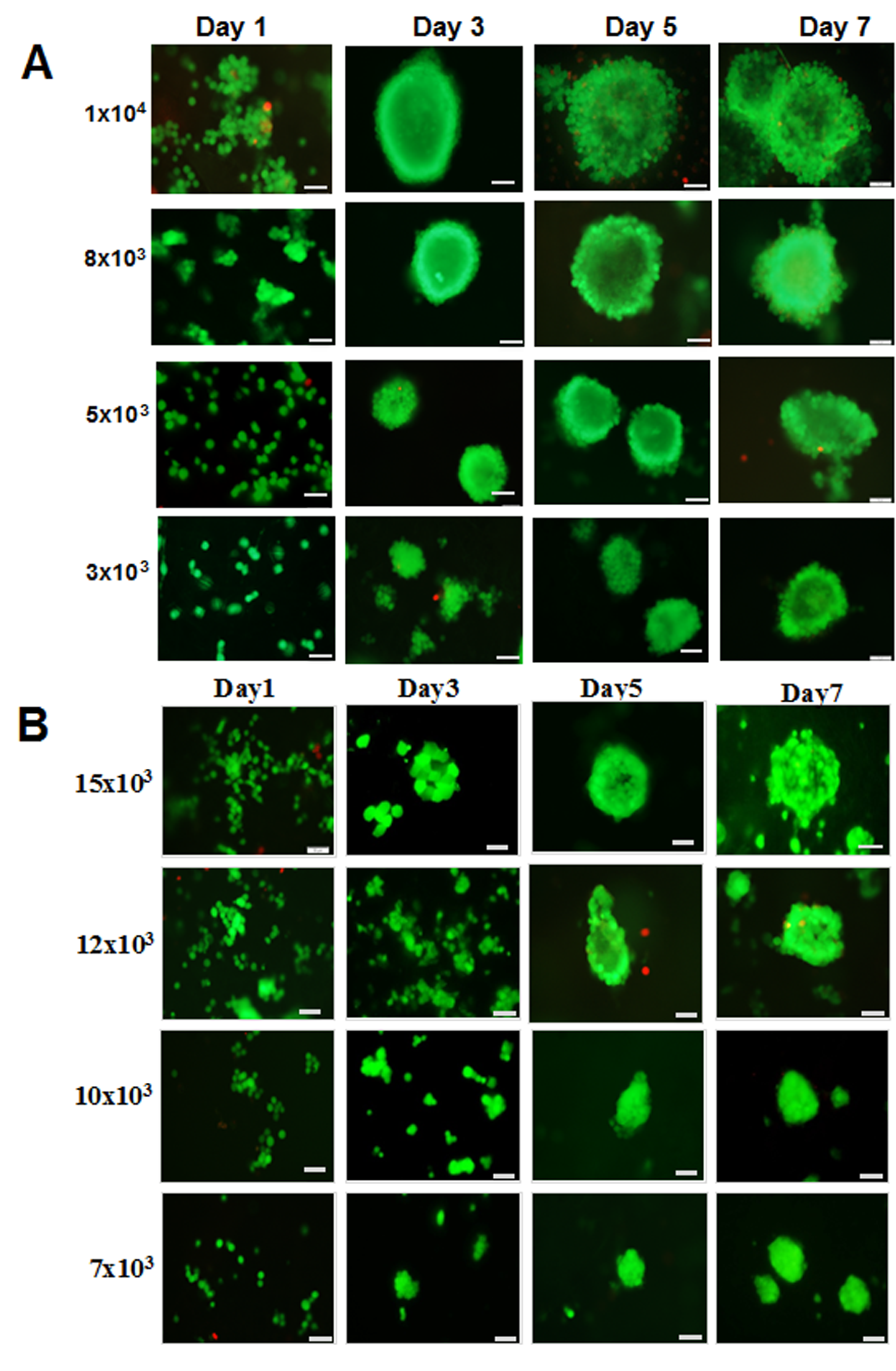

Supplement: Figure S2 — The relationship between seeding density and tumoroid formation. (A) LLC1 cells were cultured at concentrations ranging from 3×10^3^ to 1×10^4^. (B) MCF7 cells were cultured at 7×10^3^ to 15×103. Cells were stained with calcein AM/EthD-1 to detect live (green) and dead (red) cells. Scale bar = 50 µm. (TIF) [file pone.0075345.s002.tif]

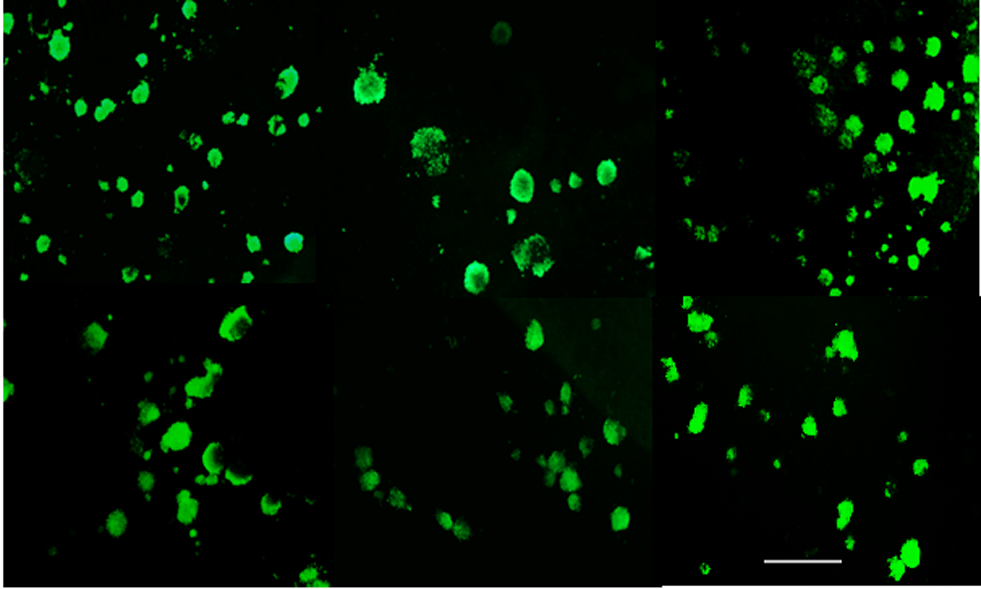

Supplement: Figure S3 — Full planar distribution of LLC1 tumoroids on 3P scaffold (day3). A representative composite picture of all sectors of the scaffold viewed using a10× lens. Scale bar = 500 µm. (TIF) [file pone.0075345.s003.tif]

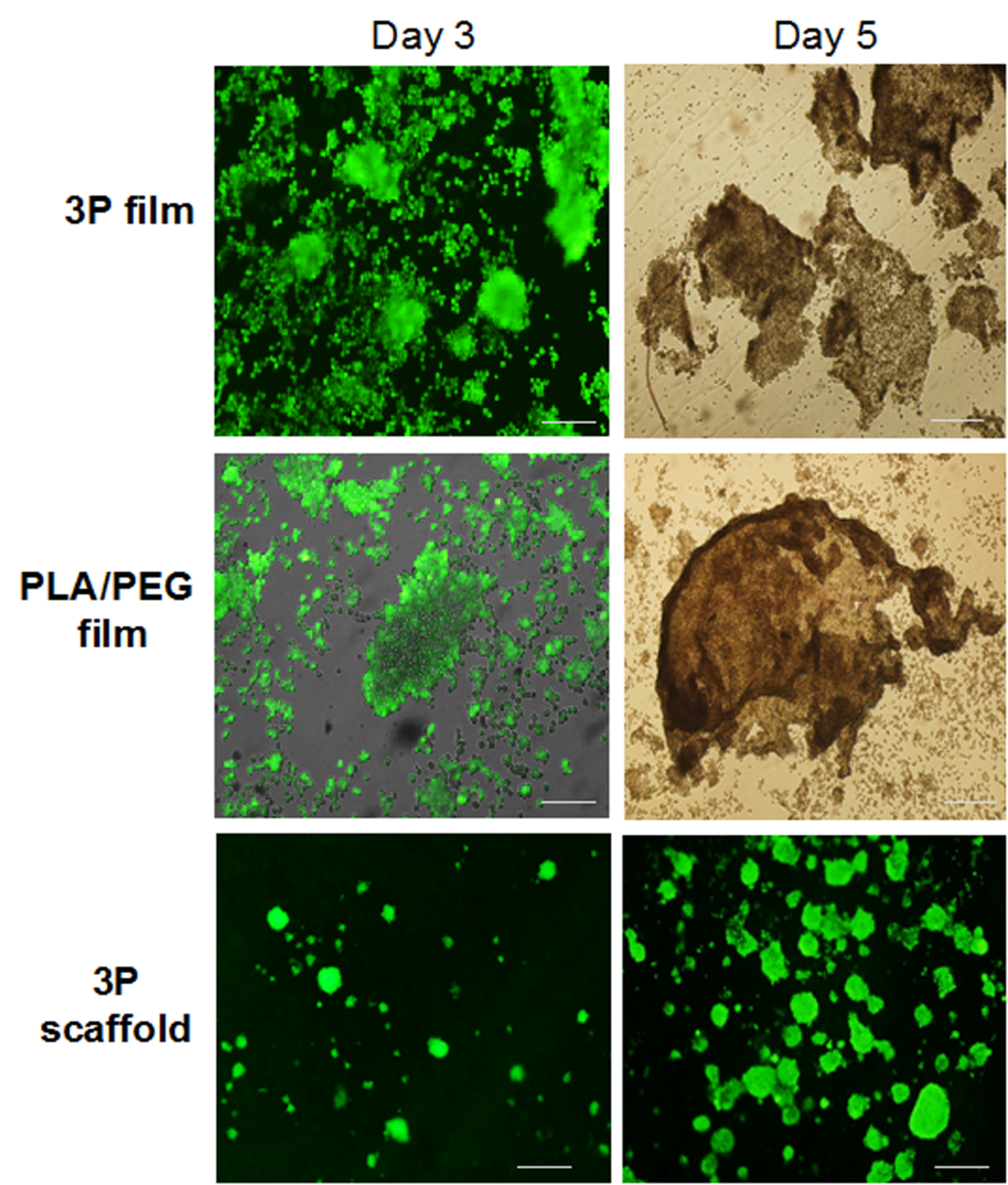

Supplement: Figure S4 — Effects of topography on tumoroid formation. LLC1 cells (5×10^4^) were cultured on 3P and mPEG-PLA films and on 3P scaffold (5×10^3^) from days 3–5. Cells were stained with calcein AM/EthD-1 to detect live (green) and dead (red) cells. Scale bar = 500 µm. (TIF) [file pone.0075345.s004.tif]

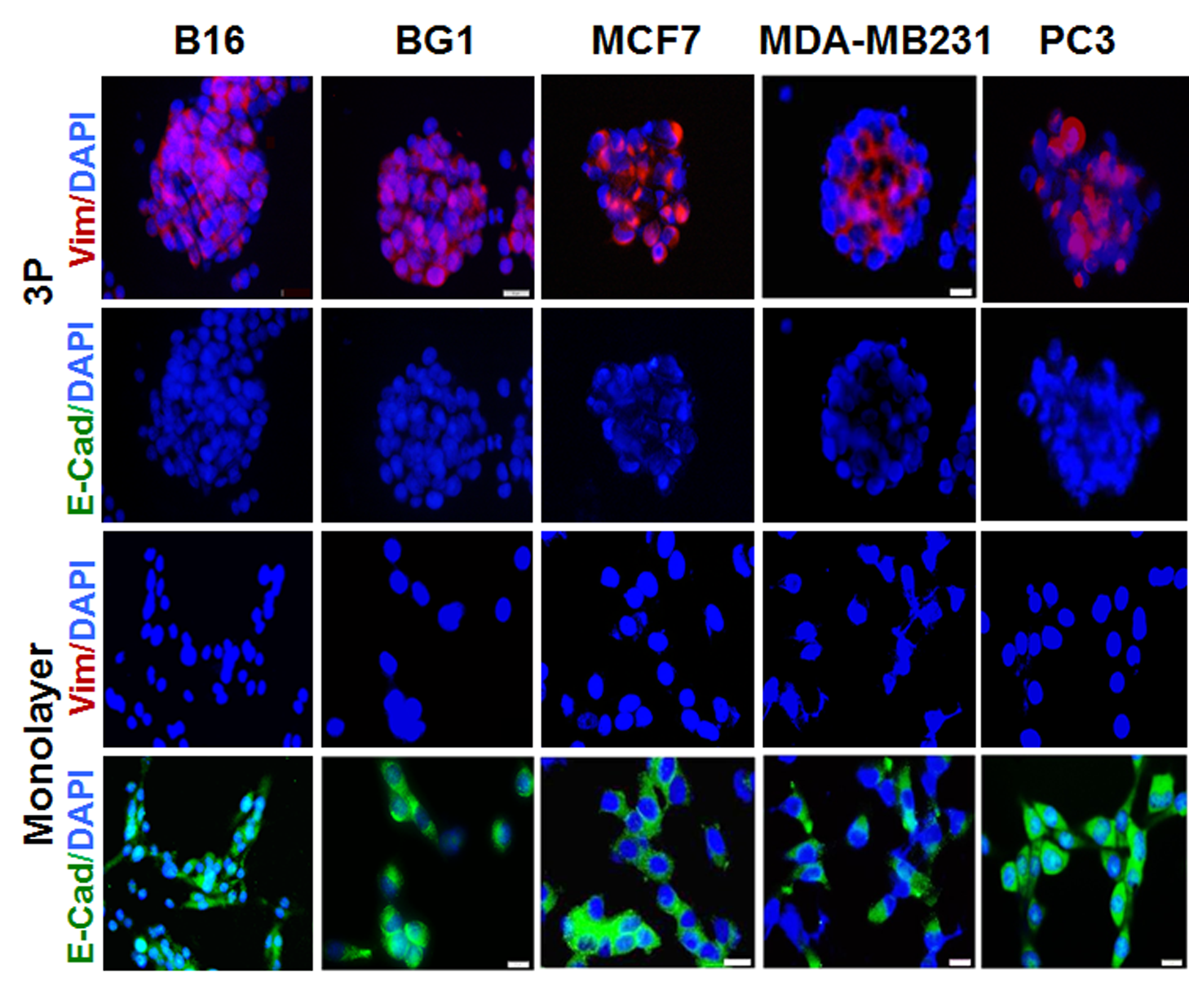

Supplement: Figure S5 — Cancer cells grown on 3P scaffold showed EMT. B16, BG1, MCF7, MDA-MB231 and PC3 cells cultured on 3P scaffolds or monolayer, fixed and immunostained with anti-E-cadherin (green), anti-vimentin (red) and DAPI (blue) for staining nuclei. Scale bar = 20 µm. (TIF) [file pone.0075345.s005.tif]

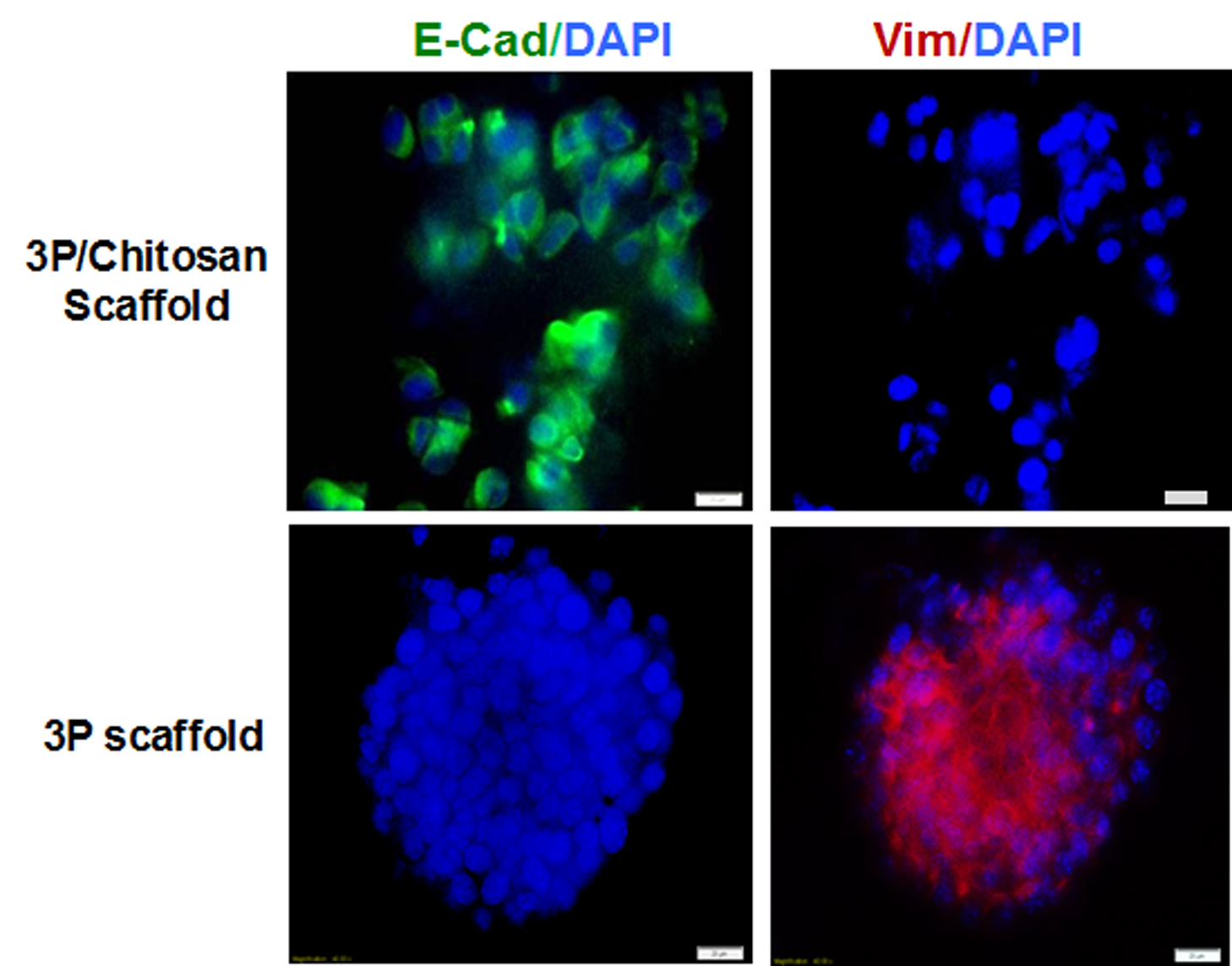

Supplement: Figure S6 — LLC1 grown on 3P/chitosan composite scaffold failed to show EMT. LLC1 cells cultured on 3P/chitosan composite scaffold or 3P scaffold, fixed and immunostained with anti-E-cadherin (green), anti-vimentin (red) and DAPI (blue) for staining nuclei. Scale bar = 20 µm. (TIF) [file pone.0075345.s006.tif]
